# RHYTHM-AF: design of an international registry on cardioversion of atrial fibrillation and characteristics of participating centers

**DOI:** 10.1186/1471-2261-12-85

**Published:** 2012-10-02

**Authors:** Harry JGM Crijns, Lori D Bash, François Chazelle, Jean-Yves Le Heuzey, Thorsten Lewalter, Gregory YH Lip, Aldo P Maggioni, Alfonso Martín, Piotr Ponikowski, Mårten Rosenqvist, Prashanthan Sanders, Mauricio Scanavacca, Alexandra A Bernhardt, Sreevalsa Unniachan, Hemant M Phatak, Anselm K Gitt

**Affiliations:** 1 Maastricht University Medical Center, Maastricht, Netherlands; 2 Merck & Co., Inc, Whitehouse Station, Readington, NJ, USA; 3 Merck, Sharpe & Dohme, Paris, France; 4 European Hospital Georges Pompidou, Rene Descartes University, Paris, France; 5 University of Bonn, Bonn, Germany; 6 University of Birmingham, Birmingham, UK; 7 ANMCO Research Center, Florence, Italy; 8 University Hospital Severo Ochoa, Madrid, Spain; 9 Medical University, Wroclaw, Poland; 10 Karolinska Institutet, Stockholm, Sweden; 11 University of Adelaide, Adelaide, Australia; 12 University of Sao Paulo, Sao Paulo, Brazil; 13 Institut für Herzinfarktforschung Ludwigshafen, Ludwigshafen, Germany; 14 UMDNJ School of Public Health, Piscataway, NJ, USA

**Keywords:** Atrial fibrillation, Cardioversion, Heart failure, Stroke, Registry

## Abstract

**Background:**

Atrial fibrillation is a serious public health problem posing a considerable burden to not only patients, but the healthcare environment due to high rates of morbidity, mortality, and medical resource utilization. There are limited data on the variation in treatment practice patterns across different countries, healthcare settings and the associated health outcomes.

**Methods/design:**

RHYTHM-AF was a prospective observational multinational study of management of recent onset atrial fibrillation patients considered for cardioversion designed to collect data on international treatment patterns and short term outcomes related to cardioversion. We present data collected in 10 countries between May 2010 and June 2011. Enrollment was ongoing in Italy and Brazil at the time of data analysis. Data were collected at the time of atrial fibrillation episode in all countries (Australia, Brazil, France, Germany, Italy, Netherlands, Poland, Spain, Sweden, United Kingdom), and cumulative follow-up data were collected at day 60 (±10) in all but Spain. Information on center characteristics, enrollment data, patient demographics, detail of atrial fibrillation episode, medical history, diagnostic procedures, acute treatment of atrial fibrillation, discharge information and the follow-up data on major events and rehospitalizations up to day 60 were collected.

**Discussion:**

A total of 3940 patients were enrolled from 175 acute care centers. 70.5% of the centers were either academic (44%) or teaching (26%) hospitals with an overall median capacity of 510 beds. The sites were mostly specialized with anticoagulation clinics (65.9%), heart failure (75.1%) and hypertension clinics (60.1%) available. The RHYTHM-AF registry will provide insight into regional variability of antiarrhythmic and antithrombotic treatment of atrial fibrillation, the appropriateness of such treatments with respect to outcomes, and their cost-efficacy. Observations will help inform strategies to improve cardiovascular outcomes in patients with atrial fibrillation.

**Trial registration:**

Clinical trials NCT01119716

## Background

Atrial fibrillation (AF) is the most common arrhythmia causing significant health care burden due to its growing prevalence [1-3]. Studies done in Europe and the United States have reported that the number of patients with AF is expected to nearly triple in the next four decades [4,5]. AF is linked with all-cause mortality, heart failure and stroke [6,7]. Apart from the high clinical burden, AF also is associated with a substantial economic burden because of high rates of hospitalization and other health resource use. [8-10] Little is known about the variation and frequency of administration of each type of cardioversion, and the burden of managing their related negative consequences in different countries and regions.

Pharmacological or electrical cardioversion (PCV, ECV) alleviate symptoms of AF acutely, and with successful maintenance of sinus rhythm, quality of life improves in symptomatic patients [11]. Since the early 1960s, ECV was established as treatment of choice for the termination of AF [12]. It is proven to be more effective than PCV, especially in persistent AF [13]. However, ECV can be time-consuming and resource heavy. In most clinical practices it requires support from an anesthesiologist, and caution must be taken to avoid subsequent complications including stroke, acute heart failure and ventricular fibrillation [14]. PCV-including the pill-in-the-pocket approach-is a frequently performed method of treating patients with paroxysmal AF [15]. Comparable to ECV, careful preparation is needed to avoid complications including heart failure, bradycardia and ventricular arrhythmias including Torsades de Pointes.

The 2010 European Society of Cardiology (ESC) guidelines [16] advocate more of an individualized approach to treatment, based on duration and changes in the pattern of AF. The guidelines emphasize correcting sinus rhythm early in the course of management of disease. The use of different modes of cardioversion varies by region, patient profile and the health care setting. Even among the existing studies of AF, there is a lack of clarity with regard to the variability of cardioversion strategies and practice patterns between countries, patients and settings among patients with recent onset AF.

The RHYTHM-AF study was initiated among patients with AF considered for cardioversion to document current practice of in-hospital treatment and associated short-term (60 days) complications. We describe the rationale, design and initiation of RHYTHM-AF.

## Methods

### Design

RHYTHM-AF was a prospective observational study fielded in 10 countries: Australia, Brazil, France, Germany, Italy, Netherlands, Poland, Spain, Sweden and the United Kingdom. Patients with recent onset AF considered for cardioversion were enrolled from participating hospitals and acute care centers between May 2010 and June 2011. Data were collected at the time of AF presentation and at follow-up, which was conducted at 60 days (±10 days).

### Objectives

RHYTHM-AF aimed to describe and compare treatment patterns and short term outcomes related to cardioversion of recent onset AF in the acute care setting. The study documents clinical practice patterns and relevant health outcomes globally, providing an overview of patient’s clinical characteristics and details of their treatment as related to AF. In so doing, the success rate of different modes of cardioversion and determinants of rhythm outcomes were estimated along with acute and short term (60 days) vascular outcomes as related to patient clinical characteristics, mode of cardioversion and level of adherence to the AF guidelines, and the appropriateness of anticoagulation treatments prior to and following cardioversion. From these, we assessed the burden of AF on the health care system and compared this burden between patients and regions.

### Site and patient selection

Sites were selected aiming to have adequate heterogeneity with balance in size (as gauged from the number of beds and frequency of procedures), nature (namely teaching/academic or non-academic), availability of specialty care units, and the location of the institution (rural, urban, distribution across region of each country). Centers were selected to be representative of those treating AF in participating countries.

All patients at least 18 years old with documented AF as confirmed by electrocardiogram and in whom a cardioversion was one of the planned therapeutic options were considered for the study. These included patients in whom actions were undertaken in anticipation of cardioversion (e.g. scheduled cardioversion, anticoagulation, oral loading) and for whom informed consent was obtained. Only patients who were already enrolled in the current trial, otherwise enrolled in a separate trial, and patients with atrial flutter were excluded.

### Data collection

All patient data were collected via a remote web based data collection form using the multilingual software solution EBogen©, developed by the IHF Ludwigshafen, Germany (the coordinating center for the study). The electronic case report forms (eCRF) adjusted to country-specific requirements were administered for data capture. A summary of the eCRF, including the site questionnaire detailing site characteristics, and eight sections for baseline patient and follow-up data is given in Table 1.

**Table 1:** Summary of 8 sections of eCRF and site questionnaire

**Site Questionnaire**	**Enrollment**	**Demographics**	**Information on AF**	**Medical treatment Prior to enrollment**	**Diagnostic Procedures**	**Acute Treatment of AF**	**Discharge**	**Follow**-**up at 60 days**
Center characteristics (type, size, units)	Demographics (age, gender, ethnicity)	Anthropometrics (ht, wt, bp)	Current episode (symptoms, type, time of onset, triggers)	ATT, rate control, other (type, indication, dose)	Laboratory measures (Hg, K, sCr, PG, INR-T)	AA/Rate control (type, dose),	Basics (vital status, date, time, destination)	Contact information (mode, date)
Specialties at site (clinics, physicians, number and availability, procedures available)	Admission information (date, time, site, reason)	Medical history (CVD, CHF, details thereof)	History of AF (date, frequency, nature, treatment)		ECG (rhythm, rate, PR & QT interval, QRS duration, LVH)	PCV & ECV (labs before, after, type, dose/joules & number of shocks route, worked?, time to SR, SR after)	Rhythm at discharge; LoS by unit	Basics (vital status, current rhyuthm)
Most frequent and most preferred approach to cardioversion	ECG information (date, time, results)	History of risk factors (family hx of disease, DM, smoking, htn, hyperlipidemia)			TTE (LA size, LVEDD, LV-EF, LVH)	Catheter ablation (type, technique, location, status), pacemaker, ICD implantation (type, indication)	Complications and AEs experienced (specified, date)	Recurrence and rehospitalization since discharge (date, LoS, documentation, CV info., reason)
		Other comorbidities			TEE (findings)	Surgery (time, date, location, technique)	Discharge medications (type, dose, indication)	Complications and AEs experienced (specified, date)
					Chest X-Ray, Stress test, Holter ECG, MRI, CT exam			Discharge medications (type, dose, indication)

eCRF: electronic case report form; AF: atrial fibrillation; ht: height; wt: weight; bp: blood pressure; ATT: antithrombotic treatment; Hg: mercury; K: potassium; sCr: serum creatinine; PG: plasma glucose; INR-T: International Normalized Ratio (Prothombin Time); CVD: cardiovascular disease; CHF: congestive heart failure; ECG: electrocardiogram; PCV: pharmacologic cardioversion; ECV: electrical cardioversion; SR: sinus rhythm; LoS: length of stay; DM: diabetes mellitus; TTE: transthoracic echocardiogram; LA: left atrium; LVEDD: left ventricular end diastolic diameter; LV-EF: left ventricular ejection fraction; LVH: left ventricular hypertrophy; ICD: implantable cardioverter-defibrillator; AEs: adverse events; TEE: transesophageal echocardiogram; MRI: magnetic resonance imaging.

Data were collected from all cumulative medical records created through day 60 (± 10 days) after enrollment during routine medical visits. If no visits occurred by day 60, patients received a brief telephone interview at this time. Patient vital status and information on any subsequent events, hospitalizations, or changes in treatment post index visit were recorded. If a patient could not be reached, follow-up information was collected from the patient’s next of kin.

Among the major outcomes of the study were success rate of cardioversion and recurrence of AF. A PCV procedure was considered successful if sinus rhythm or atrial rhythm with a rate < 100 beats per minute was obtained within 1 day after the start of pharmacological treatment. An ECV was defined as successful if sinus rhythm was obtained and maintained for at least 10 min after shock. Recurrence was defined as AF following successful cardioversion. It was assessed at both discharge and during follow-up.

### Study organization

A scientific committee, consisting of one representative from each country, as well as one representative from the coordinating center (11 members) had the authority to make decisions related to the design and conduct of the study as well as interpretation of the data and dissemination of the study results.

Faculty and operational personnel helped with study planning, development and execution. Broadly, scientific committee members provided content expertise, and site investigators were responsible for overseeing day-to-day operations and patient enrollment. The study was financially supported by Merck & Co., Inc. and its subsidiaries.

Consecutiveness in the present survey was strongly attempted by stressing its importance with the investigators and by tracking site by site enrollment of patients. A steady inclusion rate was considered to represent consecutiveness. Consistent monitoring of enrollment rates was conducted and upon a decrease in rate of enrollment, centers were immediately queried and prompted to continue enrollment efforts.

### Data management

Data quality assurance techniques included developing handling rules for missing or incomplete data, and range checks for critical variable as agreed upon by the investigators. Two strategies for data quality checks were implemented: front-end edits at time of data entry as well as more sophisticated quality control program that runs prior to creation of the analysis data set (including parent–child edits, consistency edits, and data transformations that will facilitate analyses).

This study was conducted in accordance with the EU Note for Guidance on Good Clinical Practice CPMP/ECH/135/95 and the Declaration of Helsinki. The study was only initiated at the site level after local and ethics approval requirements were obtained. A list of all ethics committees that provided approval can be found in the appendix.

### Statistical considerations

Sample size calculations were based on the objective to document the success rate of different cardioversion procedures. Assuming a success rate between 50 and 90 percent, a sample size of 4500 was estimated to allow the registry to approximate the success rate in the total population with a given precision of ±1.6% (range of 95%- CI: ±3.2%). This sample size would allow for estimating the success rate of different cardioversion procedures with adequate precision. Within each country, patient sample sizes were broadly based on the relative population of each country. While recruitment continued locally to meet above sample size, we report on a smaller sample that was analyzed based on globally applied data cut times. Descriptive analyses were conducted on the overall patient population as well as for each participating country. The Statistical Analysis System (SAS), release 9.2 was used for all analyses.

## Results

**Figure 1:**
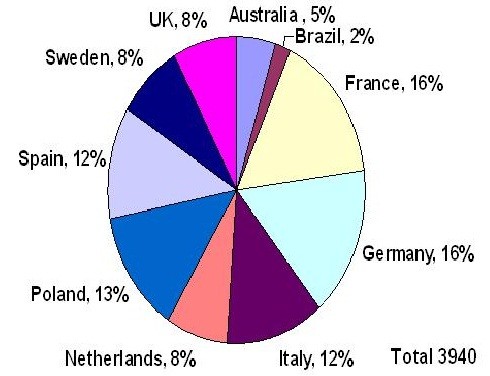
Distribution of patients per country.

A total of 175 centers were included across ten countries. Among them the Netherlands had the fewest number of sites (6) and Spain, the highest (49) (Table 2). The distribution of patients by country is illustrated in Figure 1.

**Table 2:** Hospital Characteristics by Country as Reported in the Site Questionnaires

	**Total**	**AUS**	**BR**	**FR**	**DEU**	**ITL**	**NL**	**POL**	**ESP**	**SWE**	**UK**
**Number of sites**	175	8	10	27	22	10	6	15	49	14	14
	% (**n**/**N**)	% (**n**/**N**)	% (**n**/**N**)	% (**n**/**N**)	% (**n**/**N**)	% (**n**/**N**)	% (**n**/**N**)	% (**n**/**N**)	% (**n**/**N**)	% (**n**/**N**)	% (**n**/**N**)
**Hospitals**, **n**	**175**	**8**	**10**	**27**	**22**	**10**	**6**	**15**	**49**	**14**	**14**
*Academic*/*Teaching*	70.5 (122/173)	100 (8/8)	70.0 (7/10)	40.0 (10/25)	95.5 (21/22)	20.0 (2/10)-	66.6 (4/6)	80.0 (12/15)	81.6 (40/49)	42.9 (6/14)-	85.7 (12/14)
*Non*-*academic*	29.5 (51/173)	-	30.0 (3/10)	60.0 (15/25)	4.5 (1/22)	80.0 (8/10)	33.3 (2/6)	20.0 (3/15)	18.4 (9/49)	57.1 (8/14)	14.3 (2/14)
	median (IQR)	median, IQR	median, IQR	median, IQR	median, IQR	median, IQR	median, IQR	median, IQR	median, IQR	median, IQR	median, IQR
	n*	n	n	n	n	n	n	n	n	n	n
*Total number of beds in hospital*	510 (350–880)	632 (493.5-750)	307 (239–520)	500 (350–1133)	803 (389–1000)	233 (120–450)	798 (550–994)	400 (200–726)	580 (400–918)	450 (92–605)	620 (500–1000)
	n = 171	n = 8	n = 10	n = 23	n = 22	n = 10	n = 6	n = 15	n = 49	n = 14	n = 14
*Number of beds in Cardiology department*	38.5 (20–65)	30(17.5-39)	27 (12–70)	57 (40–80)	81 (70–100)	18 (15–30)	53 (24–60)	46(37–80)	30 (18–40)	22 (11–36)	32 (20–80)
	n = 172	n = 8	n = 10	n = 24	n = 22	n = 10	n = 6	n = 15	n = 49	n = 14	n = 14
*Number of beds in CCU*/*ICU*	14 (8–25)	25 (13–43.5)	29 (14–37)	12 (8–15.5)	20 (12–30)	6 (0–10)	20 (12–27)	10 (7–21)	15 (11–30)	8 (4–14)	10 (8–18)
	n = 172	n = 8	n = 10	n = 24	n = 22	n = 10	n = 6	n = 15	n = 49	n = 14	n = 14
*Number of beds in EP*	0 (0–9)	0	2 (0–6)	11 (0–19)	11 (0–30)	0 (0–3)	6 (0–16)	2 (0–10)	0 (0–3)	0 (0–0)	0 (0–6)
	n = 172	n = 8	n = 10	n = 24	n = 22	n = 10	n = 6	n = 15	n = 49	n = 14	n = 14
*Number of beds in Cardiac surgery dept*.	0 (0–25)	17 (10.5-25)	10 (0–24)	15 (0–35)	11 (0–50)	0 (0–16)	31 (0–34)	10 (0–30)	0 (0–18)	0 (0–0)	0 (0–28)
	n = 171	n = 8	n = 10	n = 23	n = 22	n = 10	n = 6	n = 15	n = 49	n = 14	n = 14
*Number of Cardiologists*	11 (7–20)	12 (10.5-16)	45 (35–237)	10 (7–20)	9 (6–11)	16 (5–20)	17 (9–26)	12 (9–20)	12 (9–20)	10 (3–22)	6 (4–9)
	n = 171	n = 8	n = 10	n = 23	n = 22	n = 10	n = 6	n = 15	n = 49	n = 14	n = 14
*Number of Electrophysiologists*	2 (1–4)	3 (1–4)	5 (2–5)	3 (2–4)	2 (1–3)	4 (1–4)	4 (0–4)	4 (1–5)	4 (1–5)	1 (0–3)	1 (0–2)
	n = 171	n = 8	n = 10	n = 23	n = 22	n = 10	n = 6	n = 15	n = 49	n = 14	n = 14
*Number of Cardiac surgeons*	2 (0–6)	4 (2.5-5.5)	14 (6–33)	3 (0–4)	3 (0–8)	0 (0–11)	6 (0–7)	2 (0–6)	2 (0–6)	0 (0–0)	0 (0–6)
	n = 171	n = 8	n = 10	n = 23	n = 22	n = 10	n = 6	n = 15	n = 49	n = 14	n = 14
	% (n/N)	% (n/N)	% (n/N)	% (n/N)	% (n/N)	% (n/N)	% (n/N)	% (n/N)	% (n/N)	% (n/N)	% (n/N)
*Anticoagulation clinics*	65.9 (114/173)	25.0 (2/8)	90.0 (9/10)	20.0 (5/25)	36.4 (8/22)	80.0 (8/10)	50.0 (3/6)	53.3 (8/15)	95.9 (47/49)	78.6 (11/14)	92.9 (13/14)
*Heart failure clinics*	75.1 (130/173)	87.5 (7/8)	90.0 (9/10)	36.0 (9/25)	63.6 (14/22)	100 (10/10)	100 (6/6)	60.0 (9/15)	87.8 (43/49)	85.7 (12/14)	78.6 (11/14)
*Hypertension clinics*	60.1 (104/173)	75.0 (6/8)	90.0 (9/10)	24.0 (6/25)	45.4 (10/22)	70.0 (7/10)	33.3 (2/6)	60.0 (9/15)	89.8 (44/49)	42.9 (6/14)	35.7 (5/14)
*TEE in center*	94.8 (164/173)	100 (8/8)	100 (10/10)	100 (25/25)	100 (22/22)	90.0 (9/10)	100 (6/6)	93.3 (14/15)	91.8 (45/49)	78.6 (11/14)	100 (14/14)
*Catheter Ablation*	66.5 (115/173)	75.0 (6/8)	100 (10/10)	84.0 (21/25)	81.8 (18/22)	60.0 (6/10)	66.7 (4/6)	80.0 (12/15)	59.2 (29/49)	21.4 (3/14)	42.9 (6/14)
*Pacemaker implantation*	93.1 (161/173)	100 (8/8)	100 (10/10)	100 (25/25)	100 (22/22)	70.0 (7/10)	100 (6/6)	93.3 (14/15)	93.9 (46/49)	71.4 (10/14)	92.9 (13/14)
*Defibrillator implantation*	80.9 (140/173)	100 (8/8)	100 (10/10)	84.0 (21/25)	100 (22/22)	70.0 (7/10)	66.7 (4/6)	93.3 (14/15)	73.5 (36/49)	57.1 (8/14)	71.4 (10/14)
*Surgical Therapy for AF*	49.7 (86/173)	87.5 (7/8)	90.0 (9/10)	60.0 (15/25)	50.0 (11/22)	50.0 (5/10)	66.7 (4/6)	46.7 (7/15)	40.8 (20/49)	14.3 (2/14)	42.9 (6/14)

*AUS*, Australia; BR, Brazil; FR, France; *DEU*, Germany; *ITL*, Italy: *NL*, Netherlands; *POL*, Poland; *ESP*, Spain; *SWE*, Sweden; *UK*, United Kingdom; *CCU*, cardiac care unit; *ICU*, intensive care unit; *EP*, electrophysiology; *TEE*, transesophageal echocardiogram.

**Figure 2:**
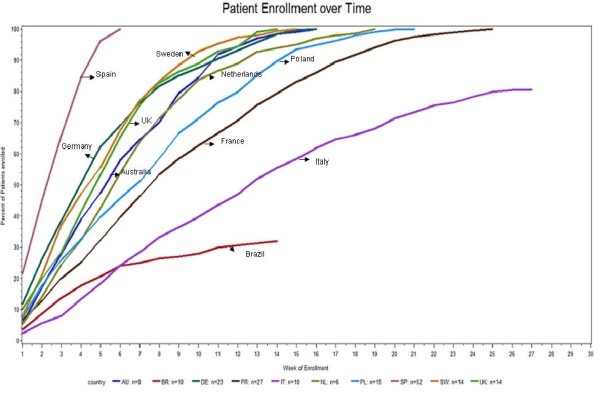
**Patient enrollment over time in participating countries.** Figure 2- the curves are normalized for time of start of recruitment.

The enrollment period prior to the global data cut in June 2011 varied from as little as 6 weeks in Spain to nearly 27 weeks in Italy. The percentage of patients enrolled over time in the different countries is highlighted in Figure 2. Of note, Brazil and Italy were still recruiting to meet their local targets at the time of the global data cut.

**Figure 3:**
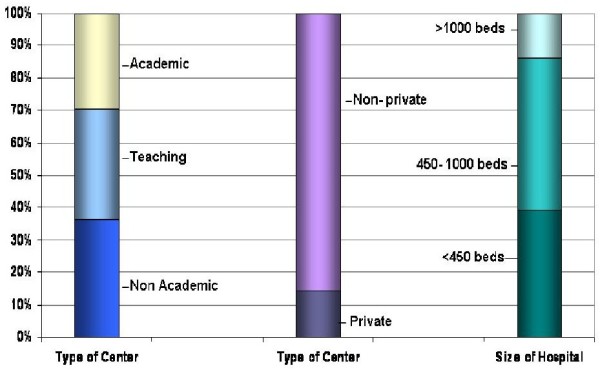
Participating hospital characteristics.

Overall, most (70.5%) centers were academic or teaching hospitals (Figure 3). This trend was consistent among most with the exceptions of Italy, France, and Sweden where non-academic sites made up 80%, 60% and 57% of all sites, respectively.

Hospitals tended to be large, with an overall median capacity of 510 beds, though there was a fairly wide distribution, with an interquartile range of 350–880 beds (Table 2). Italy had the smallest centers, with a median size of only 233 (120–450) beds, and Germany, the largest with a median of 803(389–1000) beds. The range in hospital sizes were quite wide in Germany (IQR: 389–1000), as well as in France, (IQR:350–1133). Across all sites, cardiology departments had a reasonable capacity with an overall median of 39 (20–65) beds, and CCU/ICUs with a capacity less than half that with a median of 14 beds.

Most centers had specialized anticoagulation clinics (65.9%), heart failure (75.1%) and hypertension clinics (60.1%) available, although the distribution within countries varied substantially (Table 2). France was the least equipped in each of these specialized clinics, with only 20%, 36% and 24% having anticoagulation, heart failure and hypertension clinics, respectively. Spain had the greatest availability of both anticoagulation (96%) and hypertension (90%) clinics, though Italy and the Netherlands had better availability of heart failure clinics (available at all sites versus 88% in Spain).

**Figure 4:**
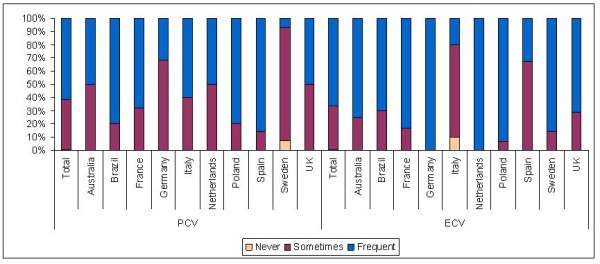
**Distribution of the percentages of centers per country reporting to use PCV or ECV** “**never**,” “**sometimes**,” **or** “**frequently**” **as reported in site questionnaires.**

The site questionnaire gauged the centers’ preferences for mode of cardioversion by asking whether one or the other type of CV was used ‘never’, ‘sometimes’, or ‘frequently’ (Table 3). Most sites (70%) indicated ‘sometimes’ using the wait-and-see approach, with only very few adopting this as the main strategy. Figure 4 shows the distribution of the 3 preference levels for PCV or ECV, by country. Sites in Sweden and Germany had a strong preference for ECV whilst avoiding PCV, whereas Italy and Spain adopted PCV as the main strategy. In the remaining countries more than 50% of sites claim to use both strategies ‘frequently’. Over 80% of all Polish centers ‘frequently’ use both forms of CV.

**Table 3:** Cardioversion Strategies by Country as Reported in the Site Questionnaires

	**Total**	**AUS**	**BR**	**FR**	**DEU**	**ITL**	**NL**	**POL**	**ESP**	**SWE**	**UK**
	**% (n/N)**	**% (n/N)**	**% (n/N)**	**% (n/N)**	**% (n/N)**	**% (n/N)**	**% (n/N)**	**% (n/N)**	**% (n/N)**	**% (n/N)**	**% (n/N)**
**Frequency** “**wait**-**and**-**see**” **approach**											
*never*	17.9 (31/173)	12.5 (1/8)	20.0 (2/10)	12.0 (3/25)	4.6 (1/22)	0	0	20.0 (3/15)	34.7 (17/49)	0	28.6 (4/14)
*sometimes*	69.4 (120/173)	75.0 (6/8)	50.0 (5/10)	60.0 (15/25)	90.9 (20/22)	80.0 (8/10)	100 (6/6)	66.7 (10/15)	59.2 (29/49)	92.9 (13/14)	57.1 (8/14)
*frequent*	12.7 (22/173)	12.5 (1/8)	30.0 (3/10)	28.0 (7/25)	4.6 (1/22)	20.0 (2/10)	0	13.3 (2/15)	6.1 (3/49)	7.1 (1/14)	14.3 (2/14)
**Frequency PCV**											
*never*	0.6 (1/173)	0	0	0	0	0	0	0	0	7.1 (1/14)	0
*sometimes*	37.6 (65/173)	50.0 (4/8)	20.0 (2/10)	32.0 (8/25)	68.2 (15/22)	40.0 (4/10)	50.0 (3/6)	20.0 (3/15)	14.3 (7/49)	85.7 (12/14)	50.0 (7/14)
*frequent*	61.8 (107/173)	50.0 (4/8)	80.0 (8/10)	68.0 (17/25)	31.8 (7/22)	60.0 (6/10)	50.0 (3/6)	80.0 (12/15)	85.7 (42/49)	7.1 (1/14)	50.0 (7/14)
**Frequency ECV**											
*never*	0.6 (1/172)	0	0	0	0	10.0 (1/10)	0	0	0	0	0
*sometimes*	32.6 (56/172)	25.0 (2/8)	30.0 (3/10)	16.7 (4/24)	0	70.0 (7/10)	0	6.7 (1/15)	67.4 (33/49)	14.3 (2/14)	28.6 (4/14)
*frequent*	66.9 (115/172)	75.0 (6/8)	70.0 (7/10)	83.3 (20/24)	100 (22/22)	20.0 (2/10)	100 (6/6)	93.3 (14/15)	32.6 (16/49)	85.7 (12/14)	71.4 (10/14)
Preferred drugs for PCV*											
*Amiodarone*	85.7 (150/175)	100 (8/8)	100 (10/10)	81.5 (22/27)	90.9 (20/22)	60.0 (6/10)	83.3 (5/6)	93.3 (14/15)	85.7 (42/49)	64.3 (9/14)	100 (14/14)
*Beta*-*blocker*	23.4 (41/175)	12.5 (1/8)	40.0 (4/10)	0	40.9 (9/22)	10.0 (1/10)	16.7 (1/6)	53.3 (8/15)	12.2 (6/49)	28.6 (4/14)	50.0 (7/14)
*Dronedarone*	8.6 (15/175)	0	0	0	45.5 (10/22)	0	0	0	0	21.4 (3/14)	14.3 (2/14)
*Flecainide*	60.6 (106/175)	75.0 (6/8)	0	33.3 (9/27)	81.8 (18/22)	60.0 (6/10)	100 (6/6)	0	91.8 (45/49)	35.7 (5/14)	78.6 (11/14)
*Propafenone*	26.9 (47/175)	0	70.0 (7/10)	3.7 (1/27)	27.3 (6/22)	80.0 (8/10)	0	80.0 (12/15)	20.4 (10/49)	7.1 (1/14)	14.3 (2/14)
*Sotalol*	12.0 (21/175)	75.0 (6/8)	20.0 (2/10)	0	0	10.0 (1/10)	33.3 (2/6)	26.7 (4/15)	2.0 (1/49)	7.1 (1/14)	28.6 (4/14)
*Verapamil*	6.3 (11/175)	12.5 (1/8)	10.0 (1/10)	0	0	10.0 (1/10)	0	13.3 (2/15)	8.2 (4/49)	0	14.3 (2/14)
*Other*†	5.1 (9/175)	0	0	0	0	10.0 (1/10)	33.3 (2/6)	0	6.1 (3/49)	14.3 (2/14)	7.1 (1/14)
**Preferred type of ECV**											
*Mono*	5.8 (10/173)	0	10.0 (1/10)	12.0 (3/25)	4.6 (1/22)	10.0 (1/10)	0	0	6.1 (3/49)	7.1 (1/14)	0
*Biphasic*	94.2 (163/173)	100 (8/8)	90.0 (9/10)	88.0 (22/25)	95.5 (21/22)	90.0 (9/10)	100 (6/6)	100 (15/15)	93.9 (46/49)	92.9 (13/14)	100 (14/14)
**Preferred heart rate target with rate control**:											
≤ *80 beats per minute*	72.5 (124/171)	100 (8/8)	40.0 (4/10)	87.5 (21/24)	95.5 (21/22)	90.0 (9/10)	66.7 (4/6)	86.7 (13/15)	55.1 (27/49)	57.1 (8/14)	69.2 (9/13)
≤ *110 beats per minute*‡	25.1 (43/171)	0	60.0 (6/10)	12.5 (3/24)	4.5 (1/22)	10.0 (1//10)	33.3 (2/6)	13.3 (2/15)	36.7 (18/49)	42.9 (6/14)	30.8 (4/13)
	**median**, **IQR**	**median**, **IQR**	**median**, **IQR**	**median**, **IQR**	**median**, **IQR**	**median**, **IQR**	**median**, **IQR**	**median**, **IQR**	**median**, **IQR**	**median**, **IQR**	**median**, **IQR**
	**n**§	**n**	**n**	**n**	**n**	**n**	**n**	**n**	**n**	**n**	**n**
**No**. **ECVs**/**year**	100 (50–200)	105 (90–200.5)	30 (9–70)	120 (85–200)	300 (150–500)	85 (15–200)	225 (115–300)	70 (40–120)	50 (24–126.5)	225 (80–280)	111(100–168)
	n = 170	n = 8	n = 10	n = 23	n = 22	n = 10	n = 6	n = 15	n = 48	n = 14	n = 14
**No**. **PCVs**/ **year**	100 (37.5-200)	102 (32.5-185)	50 (20–80)	150 (30–200)	60 (25–100)	175 (100–250)	108 (50–200)	150 (40–250)	121 (98–300)	15 (4–30)	68 (30–140)
	n = 168	n = 8	n = 9	n = 22	n = 21	n = 10	n = 6	n = 15	n = 48	n = 14	n = 14
*Number of ablation procedures* (*among those performing ablations*)	156 (53–300)	46 (35–179)	64 (20–180)	300 (112–450)	300 (155–400)	210 (120–480)	440 (279–669)	105 (53–171)	125 (40–188)	250 (120–856)	35 (19–88)
	n = 112	n = 6	n = 10	n = 19	n = 18	n = 6	n = 4	n = 12	n = 29	n = 3	n = 5

*PCV*, pharmacologic cardioversion; *ECV*, electrical cardioversion.

*Preferred drugs for PCV- sites had the option to choose one or more of drug options given, in addition to other.

†Other does not include bepridil, cibenzoline, cordichin, moricizine, tocainide which were “0” for all.

‡ ≤ 110 beats per minute: bpm: > 80 and ≤ 110.

§n = number of sites.

Amiodarone was reported as the preferred drug for pharmacological CV by sites most frequently (>85% of hospitals, Table 3), with little variation between countries. Following amiodarone, sites preferred flecainide (61%), propafenone (27%) and beta-blockers (23%) for PCV. Within countries, the preferred use of flecainide was often mutually exclusive from the preferred use of propafenone (Australia, Brazil, Netherlands, Poland). In a minority of hospitals, drugs which do not exert a direct atrial antiarrhythmic effect like the typical rate control drugs were reported as conversion drugs (Table 3). Strict rate control (target rate < 80 beats/min at rest, 72.5% of all sites) rather than lenient control was reported as main endpoint of rate control treatment by most sites. Strict rate control was relatively frequently reported in Brazil, Sweden, Poland and the Netherlands.

## Discussion

RHYTHM-AF was a large prospective multinational registry and provides a unique prospective snapshot of cardioversion practices and short term clinical outcomes across Europe, Australia and Brazil. Rhythm control by cardioversion is an important treatment modality especially for symptomatic patients with recent onset AF. There are several management options for acute or recent onset AF, but the broad picture of how patients are managed with acute rate control in clinical practice has not been extensively studied [17]. On the basis of our inventory through a questionnaire it appears that the cardioversion strategies vary considerably in the 10 countries studied. Most significant variations relate to type of conversion (PCV versus ECV versus mixed cardioversion countries) and the implementation of cardioversion (with differences in the relative frequency and of cardioversion and preferred conversion drugs). The inclusion rate was fairly consistent between countries (Figure 2), though constant rates within countries were not expected or demonstrated.

The questionnaire allowed sites to opt for more than one preferred antiarrhythmic drug therapy (since treatment preference may vary depending on patients' profiles). Amiodarone was well represented, though this agent usually does not provide rapid cardioversion. Nevertheless, it may relieve symptoms early on after start of infusion through rate control. The latter also holds for sotalol and the typical rate control drugs. In addition, amiodarone and sotalol, when used orally, may cause conversion to sinus rhythm at similar rates, i.e. between 18 and 27% of patients after 1 month of oral treatment and may therefore be seen as “wait-and-see” drugs [18,19]. Class 1C drugs are also well represented as preferred drug. The mutual exclusivity demonstrated in preferences for flecainide and propafenone among some countries may be indicative of differences in marketing activities and/or past/current availability of the drugs in these respective countries. Only a minority of hospitals across all participating countries reported use of beta-blockade for conversion, not surprising as these agents mainly control heart rate rather than prompt conversion through direct antiarrhythmic action (Figure 3). Overall, adoption of the “wait-and-see approach” did not differ substantially between countries; only a few considered this appropriate for subsets of their patients. Results suggest current practice of cardioversion uses true antiarrhythmic drugs rather than rate control drugs, reflecting a goal of early cardioversion. On the other hand, amiodarone is very frequently preferred, which may relate to the fact that patients in whom cardioversion is considered frequently harbor significant underlying heart disease, including coronary disease and heart failure. Amiodarone would indeed be reserved for the sicker patients in need of cardioversion but in whom an early and acute conversion is not required. The eventual data from RHYTHM-AF will shed more light on these questions.

Interestingly, 70% of sites overall used a stringent rate control target, as opposed to what the current ESC guidelines advise. This also varied significantly per country, i.e. between 40 and 100%, the ‘stringent countries’ being Australia, Germany, Italy, and Poland (all >80% of practices chose stringent control). It is anticipated that rate control targets will become more lenient as was shown to be acceptable in the RACE-II study [20].

Almost all centers had TEE available and implanted pacemakers. Between 50 and 100% of hospitals (within each country) in this survey performed cardioverter-defibrillator implantations. Availability of electrophysiology and ablation varied between 21% and 100% of practices in the countries, averaging 66.5%. As such, there seems to be an overrepresentation of specialized AF or arrhythmia centers. On the other hand, there was a large variation in the availability of specialized clinics among countries. To what extent these differences affect patient outcomes remains to be seen in the eventual dataset of RHYTHM-AF. Recently it has been shown that use of specialized AF clinic may significantly reduce AF-related events compared to usual care [21].

As with any observational study, this study has several limitations. It was conducted in 175 different sites, across 10 different countries, with eCRFs in 3 different languages. Protocols, eCRFs and clinical language were standardized with guidance from scientific leaders with clinical expertise in their respective countries. Nonetheless, some vulnerability to variability due to differences in practice patterns, clinical training, cultural differences, environmental variability and language subtleties must be acknowledged. In addition, although sites were selected with the aim to achieve adequate heterogeneity to be representative of each country, we cannot assess to what extent country representativeness was achieved by participating sites. In addition, while the study was designed to collect prospective data of short term outcomes, relative to the course of a chronic disease such as AF, the data collected here still reflects only a small glimpse of the course of disease and health sequelae of each patient.

Notably, in some countries, the proportion of emergency departments included in the study was greater than cardiology departments, which may be indicative of local nuance rather usual practice across the globe. It is possible that inclusion of a greater number of emergency departments (as opposed to cardiology departments) in the study may also yield differential perceptions of physician preference for PCV over ECV procedures. This can also be considered a strength in this study as it provides data of previously underrepresented clinical settings that accounts for a large and increasing number of AF patients.

In a diverse environment, where there is much heterogeneity of practice, we designed and carried out a single consistent protocol. The participants were largely defined by the “intention to cardiovert” and not necessarily by individual patient characteristics. While this renders some of the patient inclusion criterion subjective and prone to the interpretations of the treating physician, this also captures a true picture of treatment practice in that patients are treated by subjective physicians who base their decisions not only on existing published guidelines, objective patient characteristics, and hospital protocols, but also on personal experience, local practice and personal preference. Differences observed, while reflecting true differences between various settings among patients who are “considered for cardioversion” may not reflect the true differences (in treatment, outcomes, etc.) among objectively clinically homogenous patients. Results from a study designed such as this needs to be interpreted with caution and in context: observations and data collected in a homogenous manner among very heterogeneous settings.

## Conclusion

The RHYTHM-AF registry will shed light on the factors that contribute to the variability and appropriateness of cardioversion and its short term results across various treatment settings, geographic regions and within various patients. Observations will help to inform future research and clinical practice to improve outcomes in AF patients.

## Appendix

Approvals for the study were received by the following:

• Australia

• Royal Adelaide Hospital, SA

• Royal Perth Hospital, WA

• Alfred Hospital, VIC

• Monash Medical Centre, VIC

• Geelong Hospital, VIC

• Princess Alexandra, QLD

• Liverpool, NSW

• Canberra Hospital, ACT

• Brazil

• Research Ethics Committee of the Institute of Cardiovascular Diseases (Comitê de Ética em Pesquisa do Instituto de Moléstias Cardiovasculares)

• Research Ethics Committee of the HCPA (Comitê de Ética e Pesquisa do HCPA)

• Research Ethics Committee of the University of São Paulo UNIFESP / EPM (Comitê de Ética em Pesquisa da Universidade de São Paulo UNIFESP/EPM)

• Research Ethics Committee of the Albert Einstein Israeli Hospital (Comitê de Ética em Pesquisa do Hospital Israelita Albert Eisntein)

• Ethics Committee for Analysis of Research Projects HCFMUSP (Comissão de Ética para Análise de Projetos de Pesquisa do HCFMUSP (CAPPesq))

• Research Ethics Committee of PUC-PR (Comitê de Ética em Pesquisa da PUC-PR)

• Ethics Committee in Research / Faculty of Medical Sciences / UNICAMP (Comitê de Ética em Pesquisa / Faculdade de Ciências Médicas / UNICAMP)

• Research Ethics Committee of the Institute of Cardiology of the Federal District (Comitê de Ética em Pesquisa do Instituto de Cardiologia do Distrito Federal)

• Ethics Committee on Human Research of the Oswaldo Cruz German Hospital (Comitê de Ética em Pesquisa em Seres Humanos do Hospital Alemão Oswaldo Cruz)

• Research Ethics Committee - CEPesq Syrian-Lebanese Hospital (Comitê de Ética em Pesquisa – CEPesq Hospital Sírio Libanês)

• France

• French Consultative Committee on Health Research Data Processing (Comité Consultatif sur le Traitement de l'Information en Matière de Recherche dans le Domaine de la Santé)

• French Data Protection Authority (Commission nationale de l'informatique et des libertés)

• Germany

• Ethics Committee of the Bavarian Medical Association (Ethik-Kommission der Bayerischen-Landesärztekammer)

• Italy

• Ethics Committee of Azienda Ospedaliera Sant'Andrea

• Ethics Committee of Azienda Ospedaliera Riuniti di Bergamo

• Ethics Committee of Azienda ULSS 12 Veneziana

• Ethics Committee of Azienda USL di Pescara

• Ethics Committee of Azienda Provinciale Servizi Santitari APSS

• Ethics Committee of ASL RM/B

• Ethics Committee of CEAS – Azienda Sanitarie dell'Umbria

• Ethics Committee of Centro Cardiologico S.P.A. Fondazione Monzino IRCS

• Ethics Committee of ASL 3 Genovese Ospedale Villa Scassi

• Ethics Committee of Ospedale di Scilla

• Netherlands

• No IRB/ethics committee approval necessary since the study was not viewed as a study in human with an intensive change in treatment; the following sites participated

• Academic Hospital Maastrich, Maastrichtt

• Isala Klinieken Zwolle, Zwolle

• Vie CurieMedical centre Nord Limburg, Venlo

• St Antonus hospital, Nieuwegein

• Universital Medical Centre Groningen, Groningen

• Refaja Hospital, Stadskanaal

• Poland

• According to Polish law, no approvals are required for epidemiological surveys; however, the following were notified about the study:

• Bioethics Commission at the Lower Silesian Chamber of Physicians and Dentists (Komisja Bioetyczna przy Dolnośląskiej Izbie Lekarskiej)

• Committee on Bioethics at the Medical University of Warsaw (Komisja Bioetyczna przy Warszawskim Uniwersytecie Medycznym)

• Bioethics Commission (Komisja Bioetyczna)

• Bioethics Committee of the Medical University of Silesia (Komisja Bioetyczna Śląskiego Uniwersytetu Medycznego)

• Ethics Committee and Oversight research on Humans and Animals CSKMSWiA (Komisja Etyki i Nadzoru nad Badaniami nad Ludźmi i Zwierzętami CSKMSWiA)

• Bioethics Committee of the Medical University (Komisja Bioetyczna Uniwersytetu Medycznego)

• Bioethics Committee at the University of Medical Sciences (Komisja Bioetyczna przy Akademii Medycznej im)

• Bioethics Commission at the Lower Silesian Chamber of Physicians and Dentists (Komisja Bioetyczna przy Dolnośląskiej Izbie Lekarskiej)

• Bioethics Commission at the Regional Medical Chamber in Warsaw (Komisja Bioetyczna przy Okręgowej Izbie Lekarskiej w Warszawie)

• Bioethics Commission at the Institute of Cardiology (Komisja Bioetyczna przy Instytucie Kardiologii)

• Bioethics Committee of the Medical University of Silesia (Komisja Bioetyczna Śląskiego Uniwersytetu Medycznego)

• Bioethics Committee of Pomeranian Medical University (Komisja Bioetyczna Pomorskiej Akademii Medycznej)

• Bioethics Commission Medical University of Warsaw (Komisja Bioetyczna Warszawski Uniwersytet Medyczny)

• Bioethics Committee for Research on Humans Medical University (Komisja Bioetyki ds. badań na ludziach Uniwersytetu Medycznego w Łodzi)

• Spain

• Hospital Universitario La Princesa de Madrid

• Hospital del Mar

• Hospital Universitario de Bellvitge

• Hospital Universitario Vall d'Hebrón

• Hospital Regional Universitario Carlos Haya

• Hospital Universitario Virgen de las Nieves

• Hospital Ntra. Sra. del Prado

• Hospital Universitario Río Ortega

• Hospital Universitario Clínic de Barcelona

• Hospital Universitario 12 de Octubre

• Hospital Universitario Fundación Alcorcón

• Hospital Universitario de Getafe

• Hospital Universitario Puerta de Hierro Majadahonda

• Hospital Universitario Ramón y Cajal

• Hospital de Crucez

• Hospital General Universitario de Alicante

• Hospital Universitario La Fe

• Galicia

• Área 9

• Sweden

• Regional Ethical Review Board (Regionala Etikprövningsnämnden), Lund

• UK

• Tayside Committee on Medical Research Ethics B, East of Scotland Research Ethics Service

## Abbreviations

AF: Atrial fibrillation; PCV: Pharmacological cardioversion; ECV: Electrical cardioversion; ESC: European Society of Cardiology; eCRF: Electronic case report form; CV: Cardioversion; TEE: Transesophageal echocardiogram.

## Competing interests

The study and manuscript were funded by Merck and Co., Inc.

Drs. Bash, Chazelle and Phatak were full time employees of Merck and Co., Inc. at the time the study was designed.

All scientific committee members received honoraria for their time spent consulting for the study by Merck & Co., Inc.

## Authors’ contributions

HJC, LDB, FC, JYHL, TL, GYL, APM, AM, PP, MR, PS, MS, HP, and AG conceived and designed the study protocol. All contributed to study conduct. AB conducted all statistical analyses. HJC, FC, LDB, and SU drafted the manuscript and all authors read and approved the final manuscript.

## Authors’ information

Hemant M Phatak's affiliation is based on time at which work was conducted.

## Pre-publication history

The pre-publication history for this paper can be accessed here:

http://www.biomedcentral.com/1471-2261/12/85/prepub

## References

[B1] Heeringa J van der Kuip DA Hofman A Kors JA van Herpen G Stricker BH Stijnen T Lip GY Witteman JC Prevalence, incidence and lifetime risk of atrial fibrillation: the Rotterdam study Eur Heart J 2006 27 949 953 16527828 10.1093/eurheartj/ehi825

[B2] Go AS Hylek EM Phillips KA Chang Y Henault LE Selby JV Singer DE Prevalence of diagnosed atrial fibrillation in adults: national implications for rhythm management and stroke prevention: the ATRIA study JAMA 2001 285 2370 2375 10.1001/jama.285.18.2370 11343485

[B3] DeWilde S Carey IM Emmas C Richards N Cook DG Trends in the prevalence of diagnosed atrial fibrillation, its treatment with anticoagulation and predictors of such treatment in UK primary care Heart 2006 92 8 1064 1070 10.1136/hrt.2005.069492 16387813 PMC1861124

[B4] Stefansdottir H Aspelund T Gudnason V Arnar DO Trends in the incidence and prevalence of atrial fibrillation in Iceland and future projections Europace 2011 13 1110 1117 10.1093/europace/eur132 21551478

[B5] Naccarelli GV Varker H Lin J Schulman KL Increasing prevalence of atrial fibrillation and flutter in the United States Am J Cardiol 2009 104 1534 1539 10.1016/j.amjcard.2009.07.022 19932788

[B6] Wachtell K Hornestam B Lehto M Slotwiner DJ Gerdts E Olsen MH Aurup P Dahlöf B Ibsen H Julius S Kjeldsen SE Lindholm LH Nieminen MS Rokkedal J Devereux RB Cardiovascular morbidity and mortality in hypertensive patients with a history of atrial fibrillation: The Losartan Intervention For End Point Reduction in Hypertension (LIFE) study J Am Coll Cardiol 2005 45 705 711 10.1016/j.jacc.2004.06.080 15734614

[B7] Miyasaka Y Barnes ME Gersh BJ Cha SS Bailey KR Seward JB Iwasaka T Tsang TS Coronary ischemic events after first atrial fibrillation: risk and survival Am J Med 2007 120 357 363 10.1016/j.amjmed.2006.06.042 17398231

[B8] Sanoski CA Clinical, economic, and quality of life impact of atrial fibrillation J Manag Care Pharm 2009 15 6 Suppl B S4 S9 19678721 10.18553/jmcp.2009.15.s6-b.4 PMC10442900

[B9] Meinertz T Kirch W Rosin L Pittrow D Willich SN Kirchhof P ATRIUM investigators Management of atrial fibrillation by primary care physicians in Germany: baseline results of the ATRIUM registry Clin Res Cardiol 2011 100 10 897 905 10.1007/s00392-011-0320-5 21533828 PMC3178025

[B10] Wong CX Brooks AG Leong DP Roberts-Thomson KC Sanders P The increasing burden of atrial fibrillation compared to heart failure and myocardial infarction: a 15-year study of all hospitalizations in Australia Arch Intern Med 2012 172 9 739 741 10.1001/archinternmed.2012.878 22782205

[B11] Hagens VE Ranchor AV Van Sonderen E Bosker HA Kamp O Tijssen JG Kingma JH Crijns HJ Van Gelder IC RACE Study Group Effect of rate or rhythm control on quality of life in persistent atrial fibrillation. Results from the Rate Control versus Electrical Cardioversion (RACE) Study J Am Coll Cardiol 2004 43 2 241 247 10.1016/j.jacc.2003.08.037 14736444

[B12] Lown B Amarasingham R Neuman J New method for terminating cardiac arrhythmias. Use of synchronized capacitor discharge JAMA 1962 182 548 555 13931298

[B13] Van Gelder IC Tuinenburg AE Schoonderwoerd BS Tieleman RG Crijns HJ Pharmacologic versus direct-current electrical cardioversion of atrial flutter and fibrillation Am J Cardiol 1999 84 9A 147R 151R 10568674 10.1016/s0002-9149(99)00715-8

[B14] Gallagher MM Yap YG Padula M Ward DE Rowland E Camm AJ Arrhythmic complications of electrical cardioversion: relationship to shock energy Int J Cardiol 2008 123 3 307 312 10.1016/j.ijcard.2006.12.014 17395302

[B15] Alboni P Botto GL Baldi N Luzi M Russo V Gianfranchi L Marchi P Calzolari M Solano A Baroffio R Gaggioli G Outpatient treatment of recent-onset atrial fibrillation with the “pill-in-the-pocket” approach N Engl J Med 2004 351 23 2384 2391 10.1056/NEJMoa041233 15575054

[B16] Camm AJ Kirchhof P Lip GY Schotten U Savelieva I Ernst S Van Gelder IC Al-Attar N Hindricks G Prendergast B Heidbuchel H Alfieri O Angelini A Atar D Colonna P De Caterina R De Sutter J Goette A Gorenek B Heldal M Hohloser SH Kolh P Le Heuzey JY Ponikowski P Rutten FH Association European Heart Rhythm European Association for Cardio-Thoracic Surgery Guidelines for the management of atrial fibrillation: the Task Force for the Management of Atrial Fibrillation of the European Society of Cardiology (ESC) Eur Heart J 2010 31 19 2369 2429 20802247 10.1093/eurheartj/ehq278

[B17] Pisters R Nieuwlaat R Prins MH Le Heuzey JY Maggioni AP Camm AJ Crijns HJ Clinical correlates of immediate success and outcome at 1-year follow-up of real-world cardioversion of atrial fibrillation: the Euro Heart Survey Europace 2012 14 666 674 Epub ahead of print 10.1093/europace/eur406 22223715

[B18] Tieleman RG Gosselink AT Crijns HJ van Gelder IC van den Berg MP de Kam PJ van Gilst WH Lie KI Efficacy, safety, and determinants of conversion of atrial fibrillation and flutter with oral amiodarone Am J Cardiol 1997 79 53 57 10.1016/S0002-9149(96)00675-3 9024736

[B19] Singh BN Singh SN Reda DJ Tang XC Lopez B Harris CL Fletcher RD Sharma SC Atwood JE Jacobson AK Lewis HD Jr Raisch DW Ezekowitz MD Sotalol Amiodarone Atrial Fibrillation Efficacy Trial (SAFE-T) Investigators Amiodarone versus sotalol for atrial fibrillation N Engl J Med 2005 352 1861 1872 10.1056/NEJMoa041705 15872201

[B20] Van Gelder IC Groenveld HF Crijns HJ Tuininga YS Tijssen JG Alings AM Hillege HL Bergsma-Kadijk JA Cornel JH Kamp O Tukkie R Bosker HA Van Veldhuisen DJ Van den Berg MP RACE II Investigators Lenient versus strict rate control in patients with atrial fibrillation N Engl J Med 2010 362 15 1363 1373 10.1056/NEJMoa1001337 20231232

[B21] Hendriks JML De Wit R Crijns HJGM Vrijhoef HJM Prins MH Pisters R Pison LAFG Blaauw Y Tieleman RG Nurse-Led care versus usual care for patients with atrial fibrillation. Results of a randomized trial of integrated chronic care versus routine clinical care in ambulatory patients with atrial fibrillation Eur Heart J 2012 in press 10.1093/eurheartj/ehs071 22453654

